# Coherent anti-Stokes Raman spectroscopy of single and multi-layer graphene

**DOI:** 10.1038/s41467-019-11165-1

**Published:** 2019-08-14

**Authors:** A. Virga, C. Ferrante, G. Batignani, D. De Fazio, A. D. G. Nunn, A. C. Ferrari, G. Cerullo, T. Scopigno

**Affiliations:** 1 Dipartimento di Fisica, Universitá di Roma, “La Sapienza”, I-00185 Roma, Italy; 2 Istituto Italiano di Tecnologia, Center for Life Nano Science @Sapienza, Roma, I-00161 Italy; 3 Istituto Italiano di Tecnologia, Graphene Labs, Via Morego 30, I-16163 Genova, Italy; 4 Cambridge Graphene Centre, Cambridge University, 9 JJ Thomson Avenue, Cambridge, CB3 OFA UK; 5 IFN-CNR, Dipartimento di Fisica, Politecnico di Milano, P.zza L. da Vinci 32, 20133 Milano, Italy

**Keywords:** Optical properties and devices, Nonlinear optics, Raman spectroscopy

## Abstract

Spontaneous Raman spectroscopy is a powerful characterization tool for graphene research. Its extension to the coherent regime, despite the large nonlinear third-order susceptibility of graphene, has so far proven challenging. Due to its gapless nature, several interfering electronic and phononic transitions concur to generate its optical response, preventing to retrieve spectral profiles analogous to those of spontaneous Raman. Here we report stimulated Raman spectroscopy of the G-phonon in single and multi-layer graphene, through coherent anti-Stokes Raman Scattering. The nonlinear signal is dominated by a vibrationally non-resonant background, obscuring the Raman lineshape. We demonstrate that the vibrationally resonant coherent anti-Stokes Raman Scattering peak can be measured by reducing the temporal overlap of the laser excitation pulses, suppressing the vibrationally non-resonant background. We model the spectra, taking into account the electronically resonant nature of both. We show how coherent anti-Stokes Raman Scattering can be used for graphene imaging with vibrational sensitivity.

## Abstract (web summary)

Coherent anti-Stokes Raman Scattering (CARS) accesses the vibrational properties of a material via nonlinear four-wave mixing (FWM); CARS in graphene has not been observed to date despite its high nonlinear third-order susceptibility. Here, the authors devised a FWM scheme to perform stimulated Raman spectroscopy in single and multi-layer graphene through CARS.

## Introduction

Single-layer graphene (SLG) has a high nonlinear third-order susceptibility: |*χ*^(3)^| ~10^−10^ e.s.u. for harmonic generation^1^ and |*χ*^(3)^| ~10^−7^ e.s.u. for frequency mixing^2^, where one electrostatic unit of charge (1 e.s.u.), in standard units (SI) is^3^: *χ*^(3)^(SI)/*χ*^(3)^ (e.s.u.) = 4*π*/(3 × 10^4^)^2^. This is up to seven orders of magnitude greater than those of dielectric materials such as silica (*χ*^(3)^ = 1.4 × 10^−14^ e.s.u.^4^). This property is due to optical resonance with interband electronic transitions^5^ and has led to the observation of gate-tunable third-harmonic generation^1,6^ and nonlinear four-wave mixing^2,7,8^ (FWM, i.e., the third-order processes whereby an electromagnetic field is emitted by the nonlinear polarization induced by three field-matter interactions^3^). FWM can be exploited for graphene imaging, with an image contrast of up to seven orders of magnitude^2^ higher than that of optical reflection microscopy^9^. However, FWM-based imaging reported to date in graphene^2^ lacks chemical selectivity and does not provide the same wealth of information brought about by the vibrational sensitivity of Raman spectroscopy^10,11^.

**Fig. 1:**
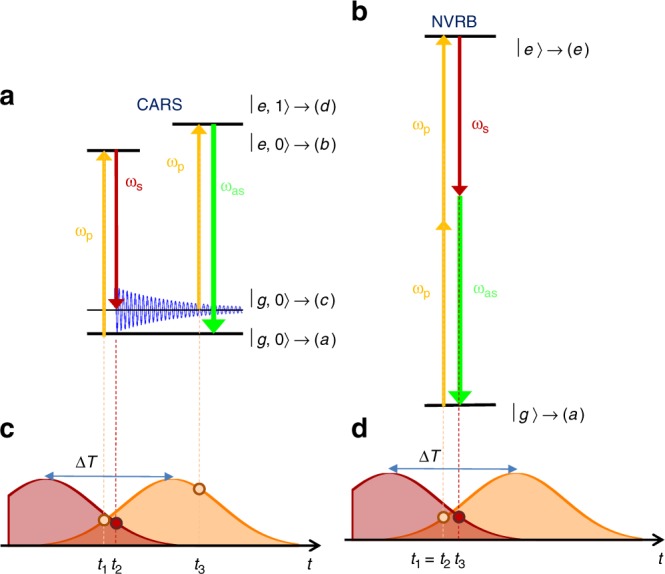
Schematic of CARS and NRVB third-order nonlinear processes. Interaction with pulses *ω*_P_, *ω*_S_, results in blue-shifted (**a**) CARS and (**b**) NVRB contributions at *ω*_as_ = 2*ω*_P_ − *ω*_S_. Since in CARS a vibrational coherence is stimulated by two consecutive interactions with the pump and Stokes fields, their frequency difference must correspond to a Raman active mode, *ω*_P_ − *ω*_S_ = *ω*_v_. **c**, **d** Constraints for the temporal sequence of the field-matter interactions (represented by circles at the top of the pulse envelopes), for CARS and NVRB. In NVRB, the three interactions generating *χ*^(3)^ happen within the few fs electronic dephasing time^20^. In CARS, the third interaction can occur over the much longer vibrational dephasing time (a few ps)^20^, within the pump pulse (PP) temporal envelope

Coherent anti-Stokes Raman scattering (CARS)^12–15^ is a FWM process that exploits the nonlinear interaction of two laser beams, the pump field *E*_P_ at frequency *ω*_P_ and the Stokes field *E*_S_ at frequency *ω*_S_ < *ω*_P_, to access the vibrational properties of a material. As shown in Fig. 1a, when the energy difference between the two photons matches a phonon energy (ℏ*ω*_P_ − ℏ*ω*_S_ = ℏ*ω*_v_), the interaction of the laser pulses and the sample results in the generation of vibrational coherences^4^, at variance with impulsive anti-Stokes spontaneous Raman (IARS) which generates vibrational population^16–19^. While spontaneous Raman (SR) scattering is an incoherent signal^20^, since the phases of the electromagnetic fields emitted by individual scatterers are uncorrelated^20^, in CARS, atomic vibrations are coherently stimulated, i.e., atoms oscillate with the same phase^4^, potentially leading to a signal enhancement of several orders of magnitude depending on incident power and scatterer density^21,22^.

The same combination of optical fields used for CARS can generate another FWM signal, a nonvibrationally resonant background (NVRB)^2^, Fig. 1b. In both processes, the optical response consists of a field emitted at the anti-Stokes frequency^4^ *ω*_as_ = 2*ω*_P_ − *ω*_S_. However, the interference of the two effects usually generates an additional contribution which is dispersive with respect to the emitted optical frequency, i.e., shaped as the first derivative of a peaked function (resembling the real part of the refractive index around a resonance), which introduces an asymmetric distortion of the Raman peak profile in the region^23^ *ω*_as_ = *ω*_P_ + *ω*_v_.

In the biological field^21,24^, a wealth of studies has demonstrated the potential of CARS for fast imaging^21,22,25^, with pixel acquisition times as low as^24^ ~0.16 μs, thus allowing for video-rate microscopy^24^. By contrast, there are only a few reports to date of CARS imaging of micro-structured materials (such as polyethylene blend^26^, multicomponent polymers^27^, and cholesterol micro-crystals^28^) and nanostructured ones (patterned gold surfaces^29^, single wall nanotubes^18,30^, and highly oriented pyrolytic graphite^31^). Such studies, performed with pixel acquisition times down to^32^ ~2 μs, have shown the ability of CARS to identify chemical heterogeneities on submicrometer scales and characterize single particles that are part of a larger domain, enabling, e.g., to visualize microscopic domains (polystyrene, polymethyl methacrylate, and polyethylene terephthalate) in the case of the above mentioned polymer mixtures^33^, or to provide detailed maps of microcrystal orientation in organic matrices (e.g., cholesterols in atherosclerotic plaques^28^).

In graphene, despite the large^1,2^ *χ*^(3)^, no CARS peak profiles, equivalent to those measured in SR, have been observed to date, to the best of our knowledge. We previously reported SR with single-color pulsed excitation^34^, using the same picosecond lasers usually adopted for CARS^24^. However, in order to measure CARS, a combination of pulses with different colors must be adopted^35^. By scanning the pulse frequency detuning in a two-color experiment, a dip has been observed^36^ in the third-order nonlinear spectral response of SLG at the G-phonon frequency. This was interpreted as an anomalous antiresonance and phenomenologically described in terms of a Fano lineshape^36^.

**Fig. 2:**
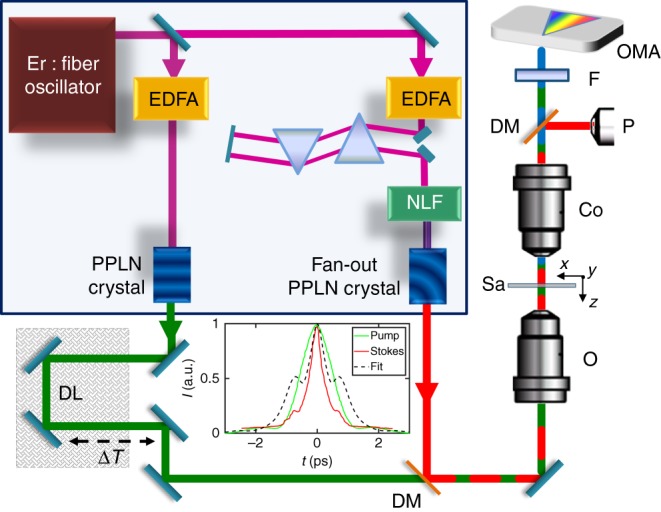
FWM setup. EDFA erbium-doped fiber amplified, NLF nonlinear fiber for SC generation, DL delay line, DM dichroic mirror, O objective, Sa sample, Co condenser, P powermeter, F filter, OMA optical multichannel analyzer. Purple, green, and red lines represent the beam pathways of 1550 nm, 784 nm (PP), and tunable SP, respectively. The second-harmonic autocorrelation of PP (green line) and SP (red line) are reported in the inset. The black dashed line simulates the autocorrelation obtained by using the profile from the fit (colored dashed lines) in Fig. 3

Here, we use two 1 ps pulses (see inset of Fig. 2) to explore FWM in SLG and few-layer graphene (FLG). We experimentally demonstrate and theoretically describe how the inter-pulse delay, Δ*T* (Fig. 1c, d) can be used to modify the relative weight of CARS and NVRB components that simultaneously contribute to the FWM, thus recovering the G-band Raman peak profile. We show that the dip in the nonlinear optical response around the vibrational resonance is due to the interplay of CARS and NVRB under electronically resonant conditions, which allows vibrational imaging with signal levels as large as those of the third-order nonlinear response.

## Results

### Sample preparation and SR characterization

SLG is grown on a 35 μm copper (Cu) foil, following the process described in ref. ^37^. The substrate is heated up to 1000 °C and annealed in hydrogen atmosphere (20 sccm) for 30 min at ~200 mTorr. Then, 5 sccm of methane (CH_4_) are let into the chamber for the following 30 min to enable growth^37,38^. The sample is then cooled back to room temperature in vacuum (~1 mTorr) and unloaded from the chamber. SLG is subsequently transferred on a glass substrate by a wet method. Polymethyl methacrylate (PMMA) is spin coated on the SLG/Cu and floated on a solution of ammonium persulfate (APS) and deionized water. When Cu is etched^38,39^, the PMMA membrane with attached SLG is then moved to a beaker with deionized water for cleaning APS residuals. The membrane is subsequently lifted with the target substrate. After drying, PMMA is removed in acetone leaving SLG on glass. SLG is characterized by SR after transfer using a Renishaw InVia spectrometer at 514 nm. The position of the G peak, Pos(G), is ~1582 cm^−1^, while FWHM(G) ~14 cm^−1^. The 2D to G peak area ratio, *A*(2D)/*A*(G), is ~5.3, indicating a p-doping after transfer ~250 meV^40,41^, which corresponds to a carrier concentration ~5 × 10^12^ cm^−^^2^. FLG flakes are produced by micromechanical cleavage from bulk graphite^42^. The bulk crystal is exfoliated on Nitto Denko tape. The FLG G peak is ~1580 cm^−1^. The D peak is negligible. The 2D peak shape indicates this is Bernal-stacked FLG^10,11^.

### CARS measurements setup

For CARS experiments, we use a two-modules Toptica FemtoFiber Pro source, with two erbium-doped fiber amplifiers at ~1550 nm generating 90 fs pulses at 40 MHz, seeded by a common mode-locked Er:fiber oscillator^43^, Fig. 2. In the first branch (FemtoFiber pro NIR), 1 ps pulses at 784 nm (pump pulse, PP) are produced by second-harmonic spectral compression^44^ in a 1 cm periodically poled lithium niobate (PPLN) crystal. In the second branch (FemtoFiber pro TNIR), the amplified laser passes through a nonlinear fiber, wherein a supercontinuum (SC) output is generated. The SC spectral intensity can be tuned with a motorized Si-prism-pair compressor. A PPLN crystal with a fan-out grating (i.e., a poling period changing along the transverse direction) is exploited to produce broadly tunable (from 840 to 1100 nm) narrowband 1 ps Stokes pulses (SP), with a power <10 mW. A dichroic mirror is used to combine the two beams, whose relative temporal delay is tuned with an optical delay line. A long-working distance 20× objective (O, numerical aperture NA = 0.4) focuses the pulses onto the sample (Sa). The Stokes power is less than 2.45 mW during the scan, while the pump power is 2 mW for SLG and 6 mW for FLG. We note that no light-induced damage of SLG occurs up to 13.5 mW as verified by SR under the same repetition rate and similar pulse durations^34^. Thus, we rule out any structural degradation by the <6 mW pulses used here. The generated and transmitted light is collected by a condenser (C) and the PP and SP are filtered out by a short-pass filter (F). The total FWM signal is collected with an optical multichannel analyzer (OMA, Photon Control SPM-002-E). A dichroic mirror reflects the SP in order to measure its intensity (*I*_s_) with a powermeter (P). FWM spectra are obtained by scanning *ω*_S_ around *ω*_P_ − *ω*_v_ (at fixed *ω*_P_) to probe the G-band phonon frequency, *ω*_v_ = *ω*_G_. To assess data reproducibility we repeated the CARS measurements (scanning *ω*_S_) finding no appreciable changes.

### CARS by time-delayed FWM

**Fig. 3:**
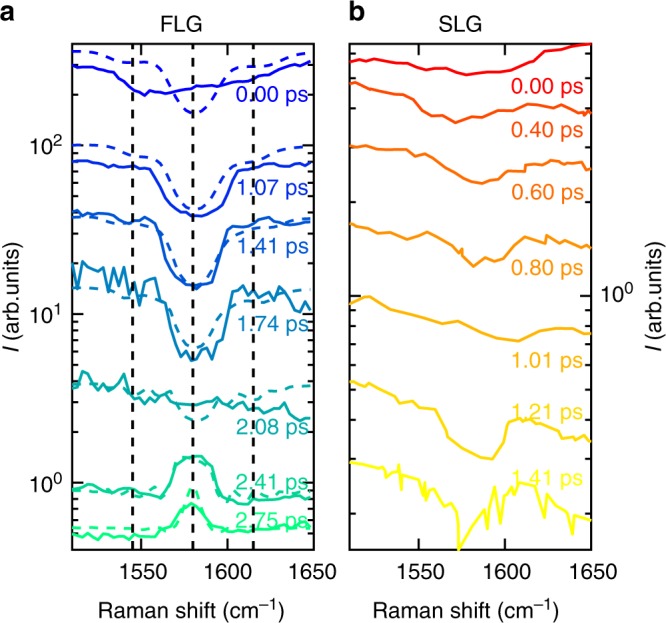
Graphene CARS spectra. CARS spectra of **a** FLG and **b** SLG as a function of Raman shift $\left( {\tilde \nu - \tilde \nu _{\mathrm{P}}} \right)$ at different Δ*T* between the beams at tunable *ω*_S_ and fixed *ω*_P_. In **a**, colored dashed lines are fits to the data using Eq. (1) and the nonlinear polarization obtained from Eqs. (19) and (20). Vertical black dashed lines indicate three energies $( {\tilde \nu _{1,2,3} - \tilde \nu _{\mathrm{P}} = 1545,1576,1607{\mathrm{cm}}^{ - 1}})$, taken as reference for the FLG CARS images in Fig. 5

Figure 3 displays the FWM intensity, normalized to *I*_s_, for different Δ*T*. In both SLG and FLG measurements, for Δ*T* shorter than the vibrational dephasing time *τ* ~1 ps^45^, i.e., the characteristic time of coherence loss^20^, a Lorentzian dip at *ω*_as_ = *ω*_P_ + *ω*_G_ appears on top of a background^36^. For Δ*T* > 2ps, while the total FWM signal decreases by nearly two orders of magnitude, the dip observed for FLG at Δ*T* ~0 ps evolves into a Raman peak shape at the G-phonon energy. No dispersive features are seen at any Δ*T*, unlike what normally expected for the interference between NVRB and CARS^23^. Here, we use pulses with duration *δt* ~ 1 ps, since this allows us to scan the inter-pulse delay across the vibrational dephasing time *τ* to suppress the NVRB cross section more than the vibrational contribution, while minimizing the spectral broadening due to the finite pulse duration $1/\delta t \sim 15\,{\mathrm{cm}}^{ - 1}$^20^.

Since both CARS and NVRB depend quadratically on the number of scatterers^33^, the SLG intensity is significantly reduced with respect to FLG (Fig. 3), with a lower signal-to-noise ratio, hampering the detection of peak-shaped vibrational resonances expected for Δ*T* > 1.4 ps.

The data in Fig. 3 can be qualitatively understood as follows. The anti-Stokes signal, *I*(*ω*_as_), generated by CARS and NVRB, is proportional to the square modulus of the electric field emitted by the third-order polarization^4^, *P*^(3)^, as:

1 $$I(\omega _{{\mathrm{as}}}) \propto |P_{{\mathrm{CARS}}}^{(3)}(\omega _{{\mathrm{as}}}) + P_{{\mathrm{NVRB}}}^{(3)}(\omega _{{\mathrm{as}}})|^2.$$

**Fig. 4:**
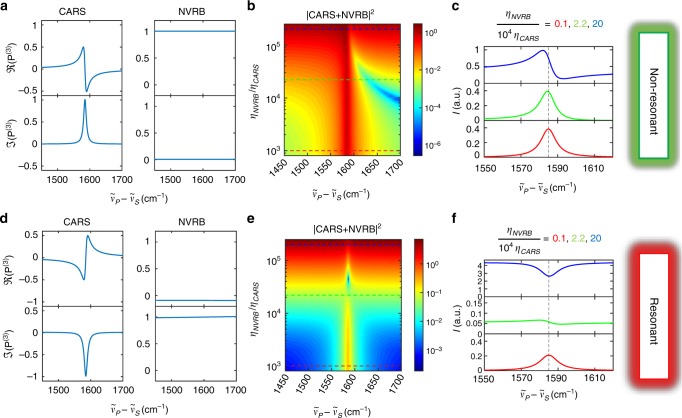
CARS and NVRB response in electronically resonant and non-resonant condition. CARS and NVRB spectral profiles for **a**–**c** electronically nonresonant and **d**–**f** resonant regimes, as derived from Eqs. (5) and (6), considering^45,47^ *τ*_*ba*_ = *τ*_*da*_ = *τ*_*ea*_ = 10 fs, *γ*_*ca*_ = FWHM(*G*)/2 = 6 cm^−1^. **a**, **c** Normalized $\Re (P_{{\mathrm{CARS}}}^{(3)})$, $\Im (P_{{\mathrm{CARS}}}^{(3)})$, and $\Re (P_{{\mathrm{NRVB}}}^{(3)})$, $\Im (P_{{\mathrm{NRVB}}}^{(3)})$. Colormaps in **b**, **e** generalize **a**, **d** for different *η*_NVRB_/*η*_CARS_, as for Eqs. (7) and (8). In **c**, **f**, selected spectra corresponding to three *η*_NVRB_/*η*_CARS_ from the colormap are reported

CARS and NVRB are simultaneously generated by two light-matter interactions with the PP and a single interaction with the SP, with different time ordering, Fig. 1a, b. Consequently, $P^{(3)} \propto E_{\mathrm{P}}^2E_{\mathrm{S}}^ \ast$, where * indicates the complex conjugate. Therefore, the FWM signals are quadratic with respect to the pump power and linear with respect to the Stokes power. However, the temporal constraints for such interactions are significantly different for the two cases. As shown in Fig. 1c, d, in the case of NVRB the three interactions must take place within the dephasing time of the involved electronic excitation, which in SLG is ~10 fs^46,47^, i.e., much shorter than the pulses duration (*δt* ~ 1 ps). Hence, $P_{{\mathrm{NVRB}}}^{(3)}$ (*ω*_as_) is only generated during the temporal overlap between the two pulses $P_{{\mathrm{NVRB}}}^{(3)} \propto E_{\mathrm{P}}^2(t - \Delta T)E_{\mathrm{S}}^ \ast (t)$ (the three field interactions, in a representative NVRB event, are indicated by three nearly coincident dots in Fig. 1d). In CARS, the electronic dephasing time only constrains the lag between the first two interactions that generate the vibrational coherence (the two stimulating-field interactions are represented by the two nearly coincident dots in Fig. 1c). This can be read out by the third field interaction within the phonon dephasing time, *τ* ~ 1 ps^45^ (indicated, for a representative CARS event, by the third dot in Fig. 1d). Thus, $P_{{\mathrm{CARS}}}^{(3)} \propto E_{\mathrm{P}}(t - {\mathrm{\Delta }}T)E_{\mathrm{S}}^ \ast (t){\int}_{ - \infty }^t E_{\mathrm{P}}(t{\prime} - {\mathrm{\Delta }}T)e^{ - t{\prime}/\tau }dt{\prime}$^48^. Therefore, Δ*T* can be used to control the relative weights of $P_{{\mathrm{CARS}}}^{(3)}$ and $P_{{\mathrm{NVRB}}}^{(3)}$^22,48–52^. For positive time delays within a few *τ*, $P_{{\mathrm{CARS}}}^{(3)}/P_{{\mathrm{NVRB}}}^{(3)}$ is progressively enhanced, as shown in Fig. 4a–c.

### Electronically resonant and non-resonant FWM

The system response can be evaluated through a density-matrix description^20^ of $P^{(3)}(\omega ,{\mathrm{\Delta }}T)$. In the presence of a temporal delay between PP and SP, their electric fields can be written as^3^: $E_{\mathrm{P}}(t,{\mathrm{\Delta }}T) = A_{\mathrm{P}}(t,{\mathrm{\Delta }}T)e^{ - i\omega _{\mathrm{P}}t}$ and $E_{\mathrm{S}}(t,0) = A_{\mathrm{S}}(t,0)e^{ - i\omega _{\mathrm{S}}t}$, where *A*_P/S_(*t*, Δ*T*) indicates the PP/SP temporal envelope. By Fourier transform, the fields can be expressed in the frequency domain as $\hat E_{\mathrm{P}}(\omega ,{\mathrm{\Delta }}T) = {\int}_{ - \infty }^{ + \infty } E_{\mathrm{P}}(t,{\mathrm{\Delta }}T)e^{i\omega t}dt$ and $\hat E_{\mathrm{S}}(\omega ,0) = {\int}_{ - \infty }^{ + \infty } E_{\mathrm{S}}(t,0)e^{i\omega t}dt$, which can be used to calculate $P_{{\mathrm{CARS}}}^{(3)}(\omega ,\Delta T)$ as^20,53^

2 $$	{P_{{\mathrm{CARS}}}^{(3)}(\omega ,{\mathrm{\Delta }}T) \propto - \eta _{{\kern 1pt} {\mathrm{CARS}}}\mathop {\int}\limits_{ - \infty }^\infty d \omega _3\mathop {\int}\limits_{ - \infty }^\infty d \omega _2\mathop {\int}\limits_{ - \infty }^\infty} {d \omega _1} \\ 	\times{{ \hat A_{\mathrm{P}}(\omega _3,{\mathrm{\Delta }}T)\hat A_{\mathrm{P}}(\omega _1,{\mathrm{\Delta }}T)\hat A_{\mathrm{S}}^ \ast (\omega _2,0)\delta (\omega - 2\omega _P + \omega _S - \omega _3 - \omega _1 + \omega _2)} \over {\left( {\omega _{\mathrm{P}} + \omega _3 - \bar \omega _{ba}} \right)\left( {\omega _{\mathrm{P}} - \omega _{\mathrm{S}} + \omega _3 - \omega _2 - \bar \omega _{ca}} \right)\left( {2\omega _{\mathrm{P}} - \omega _{\mathrm{S}} + \omega _3 - \omega _2 + \omega _1 - \bar \omega _{da}} \right)}},$$

where *η*_CARS_ = *n*_CARS _*μ*_*ba *_*μ*_*cb *_*μ*_*cd *_*μ*_*ad*_, *μ*_*ij*_ is the transition dipole moment between the *i* and *j* states, *n*_CARS_ is the number of scatterers involved in the CARS process, $\bar \omega _{ij} = \omega _{ij} - i\gamma _{ij} = \omega _i - \omega _j - i\gamma _{ij}$ is the energy difference between the levels *i* and *j*, and $\gamma _{ij} = \tau _{ij}^{ - 1}$ is the dephasing rate of the |*i*〉 〈*j*| coherence^20^; *a* and *c* denote the vibrational ground state |*g*, 0〉, and the first vibrational excited level, |*g*, 1〉, with respect to the electronic ground state |*g*〉 (*π* band). In our experiments, *c* corresponds to the G phonon at *q* ~ 0, *b* an*d d* indicate the vibrational ground stat*e*, |*e*, 0〉, and the first vibrational excited level, |*e*, 1〉, with respect to the excited electronic stat*e* |*e*〉 (π^*^ band).

Using the conservation of energy represented by the *δ*-distribution in Eq. (2) and integrating over *ω*_2_, we get:

3 $$\begin{array}{*{20}{l}} {P_{{\mathrm{CARS}}}^{(3)}(\omega ,{\mathrm{\Delta }}T)} \hfill & \propto \hfill & { - \eta _{{\mathrm{CARS}}}\mathop {\int}\limits_{ - \infty }^\infty d \omega _1\mathop {\int}\limits_{ - \infty }^\infty d \omega _3} \hfill \\ {} \hfill & {} \hfill & { \times {\textstyle{{\hat A_{\mathrm{P}}(\omega _3,{\mathrm{\Delta }}T)\hat A_P(\omega _1,{\mathrm{\Delta }}T)\hat A_{\mathrm{S}}^ \ast (2\omega _{\mathrm{P}} - \omega _{\mathrm{S}} - \omega + \omega _3 + \omega _1,0)} \over {\left( {\omega _{\mathrm{P}} + \omega _3 - \bar \omega _{ba}} \right)\left( {\omega - \omega _{\mathrm{P}} - \omega _1 - \bar \omega _{ca}} \right)\left( {\omega - \bar \omega _{da}} \right)}}}}. \hfill \end{array}$$

Defining $\tilde \nu = \omega /(2\pi c)$, the third-order nonlinear polarization can be expressed as a function of the Raman shift $\left( {\tilde \nu - \tilde \nu _{\mathrm{P}}} \right)$ as $P^{(3)}(\omega ,\Delta T) = P^{(3)}(2\pi c\tilde \nu ,{\mathrm{\Delta }}T)$.

The *ω*_*ca*_ level in the denominator of Eq. (3) is the frequency of the Raman mode coherently stimulated in CARS, while *ω*_*ba*_ and *ω*_*da*_ are frequency differences between the electronic levels. In the case of real levels, resonance enhancement occurs^20^. In view of the optical nature of the involved phonons (*q* ~ 0), and due to momentum conservation, only one electronic level must be included in the calculation and, consequently, the nonlinear response can be derived for *ω*_*ba*_ = *ω*_*dc*_ = *ω*_*P*_. In a similar manner, $P_{{\mathrm{NVRB}}}^{(3)}$ can be expressed as^20^

4 $$\begin{array}{*{20}{l}} {P_{{\mathrm{NVRB}}}^{(3)}(\omega ,{\mathrm{\Delta }}T)} \hfill & \propto \hfill & { - \eta _{{\mathrm{NVRB}}}\mathop {\int}\limits_{ - \infty }^\infty d \omega _1\mathop {\int}\limits_{ - \infty }^\infty d \omega _2} \hfill \\ {} \hfill & {} \hfill & { \times {\textstyle{{\hat A_{\mathrm{P}}(\omega _1,{\mathrm{\Delta }}T)\hat A_{\mathrm{P}}(\omega _2,{\mathrm{\Delta }}T)\hat A_{\mathrm{S}}^ \ast (2\omega _{\mathrm{P}} - \omega _{\mathrm{S}} - \omega + \omega _1 + \omega _2,0)} \over {\left( {\omega _{\mathrm{P}} + \omega _1 - \bar \omega _{ea}} \right)\left( {2\omega _{\mathrm{P}} + \omega _1 + \omega _2 - \bar \omega _{ea}} \right)\left( {\omega - \bar \omega _{ea}} \right)}}}}, \hfill \end{array}$$

where *η*_NVRB_ = *n*_NVRB_|*μ*_*ea*_|^4^, *n*_NVRB_ is the number of scatterers involved in the NVRB process, and *ω*_*ea*_ is the energy of the electronic excited level involved in the NVRB process. Since the cross section of third-order nonlinear processes in graphene is enhanced by increasing the photon energy^54,55^, we consider only the dominant case, i.e., $\tilde \nu _{ea} = 2\tilde \nu _{\mathrm{P}}$.

We describe the spectral FWM response assuming monochromatic fields with no inter-pulse delay: $\hat E_{\mathrm{P}}(\omega ) = E_{\mathrm{P}} \cdot \delta (\omega - \omega _{\mathrm{P}})$, $\hat E_{\mathrm{S}}(\omega ) = E_{\mathrm{S}} \cdot \delta (\omega - \omega _{\mathrm{S}})$. From Eqs. (3) and (4), the CARS and NVRB nonlinear polarizations can be expressed as^4,20^

5 $$\begin{array}{*{20}{l}} {P_{{\mathrm{CARS}}}^{(3)}(\omega )} \hfill & \propto \hfill & { - \frac{{\eta _{CARS}E_P^2E_S^ \ast }}{{(\omega _P - \bar \omega _{ba})(\omega - \omega _P - \bar \omega _{ca})(\omega - \bar \omega _{da})}}} \hfill \\ {} \hfill & = \hfill & {\chi _{CARS}^{(3)}E_P^2E_S^ \ast }; \hfill \end{array}$$

6 $$\begin{array}{*{20}{l}} {P_{{\mathrm{NVRB}}}^{(3)}(\omega )} \hfill & \propto \hfill & { - \frac{{\eta _{{\mathrm{NVRB}}}E_{\mathrm{P}}^2E_{\mathrm{S}}^ \ast }}{{(\omega _{\mathrm{P}} - \bar \omega _{ea})(2\omega _{\mathrm{P}} - \bar \omega _{ea})(\omega - \bar \omega _{ea})}}} \hfill \\ {} \hfill & = \hfill & {\chi _{{\mathrm{NVRB}}}^{(3)}E_{\mathrm{P}}^2E_{\mathrm{S}}^ \ast }, \hfill \end{array}$$

which can be used to calculate the total FWM spectrum according to Eq. (1). Figure 4a plots the electronically nonresonant case. The CARS polarization, defined by Eq. (5), is a complex quantity: the real part has a dispersive lineshape, while the imaginary part peaks at *ω*_*ca*_. The NVRB polarization, defined by Eq. (6), is a positive real quantity. Accordingly, the FWM spectrum in the electronically non-resonant condition, *I*(*ω*_as_)^NR^, can be written as^20^

7 $$\begin{array}{*{20}{l}} {I(\omega _{{\mathrm{as}}})^{{\mathrm{NR}}}} \hfill & \sim \hfill & {|P_{{\mathrm{NVRB}}}^{(3)}|^2 + |P_{{\mathrm{CARS}}}^{(3)}|^2 + 2\Re (P_{{\mathrm{NVRB}}}^{(3)})\Re (P_{{\mathrm{CARS}}}^{(3)})} \hfill \\ {} \hfill & \propto \hfill & {|\chi _{{\mathrm{NVRB}}}^{(3)}|^2 + |\chi _{{\mathrm{CARS}}}^{(3)}|^2 + 2\Re (\chi _{{\mathrm{NVRB}}}^{(3)})\Re (\chi _{{\mathrm{CARS}}}^{(3)})} \hfill, \end{array}$$

and it can be significantly distorted by the third term in Eq. (7) depending on the relative weight of the two corresponding susceptibilities. The maximum of the signal, when the dispersive contribution is dominant, can be frequency shifted from the phonon frequency. This is the most common scenario, with the dispersive lineshapes hampering direct access to the vibrational characterization of the sample in terms of phonon frequencies and lifetimes. Such limitation is particularly severe when $\chi _{{\mathrm{NRVB}}}^{(3)}$ is comparable to $\chi _{{\mathrm{CARS}}}^{(3)}$ and the NVRB and CARS contributions have the same order of magnitude. This condition is common in the case of a weak vibrational resonant contribution $\left( {\frac{{\mu _{ba}\mu _{cb}\mu _{cd}\mu _{ad}}}{{|\mu _{ea}|^4}} < < 1} \right)$, as in the case of low concentrations of oscillators $\left( {\frac{{n_{{\kern 1pt} {\mathrm{CARS}}}}}{{n_{{\kern 1pt} {\mathrm{NVRB}}}}} < < 1} \right)$. Hence, this produces an intense NVRB signal and reduces the vibrational contrast, hindering the imaging of electronically nonresonant samples. This is the case for cells and tissues which need to be excited in the near infrared to avoid radiation damage^56^.

For SLG, the linear dispersion of the massless Dirac Fermions makes the response always electronically resonant. In the case of FLG, absorption has a complex dependence on wavelength, as well as on the number of layers and their relative orientation, exhibiting, e.g., a tunable band gap in twisted bilayer graphene^57^. This is also reflected in the resonant nature of SR^58,59^. However, approaching visible wavelengths, the absorption spectrum flattens above ~0.8 eV and it is $\sim (1 - \pi e^2/2h)^N$ for Bernal-stacked *N*-layer graphene^60^. Our exfoliated FLG are Bernal stacked, as also confirmed by the measured 2D peak shape in SR^10,11^. Accordingly, at the typical CARS wavelengths used here (784 and 894 nm), SLG and Bernal FLG are electronically resonant, unlike the situation for most biological samples^56^. This results in an opposite sign in the CARS response, i.e., a spectral dip in $\Im (\chi _{{\mathrm{CARS}}}^{(3)})$, related to two additional imaginary unit contributions in the denominator of Eq. (5), $(\omega _{\mathrm{P}} - \bar \omega _{ba})$ and $(\omega - \bar \omega _{da})$, wherein the $i\gamma _{ba}$, $i\gamma _{da}$ components dominate. Further, the −*i* contribution from $(2\omega _P - \bar \omega _{ea})$ results in a NVRB dominated by the imaginary part, as illustrated in Fig. 4d–f.

Thus, the third term in Eq. (7) must be replaced with the contribution from the interference of the spectral dip $\Im (\chi _{{\mathrm{CARS}}}^{(3)})$ with the imaginary part $\Im (\chi _{{\mathrm{NRVB}}}^{(3)})$. This leads to a signal that, under the electronically resonant regime, becomes^20^

8 $$\begin{array}{*{20}{l}} {I(\omega _{{\mathrm{as}}})^{\mathrm{R}}} \hfill & {\sim |P_{{\mathrm{NRVB}}}^{(3)}|^2 + |P_{{\mathrm{CARS}}}^{(3)}|^2 + 2\Im (P_{{\mathrm{NRVB}}}^{(3)})\Im (P_{{\mathrm{CARS}}}^{(3)})} \hfill \\ {} \hfill & { \propto |\chi _{{\mathrm{NRVB}}}^{(3)}|^2 + |\chi _{{\mathrm{CARS}}}^{(3)}|^2 + 2\Im (\chi _{{\mathrm{NRVB}}}^{(3)})\Im (\chi _{{\mathrm{CARS}}}^{(3)})} \hfill, \end{array}$$

which indicates that the total FWM, at the phonon frequency, can be either a negative dip or a positive peak depending on the ratio between vibrationally resonant and nonresonant susceptibilities $\left( {\chi _{{\mathrm{CARS}}}^{(3)}/\chi _{{\mathrm{NRVB}}}^{(3)}} \right)$, which depends only on the material under examination and not on the pulses used in the experiment. Such a qualitatively different interplay between NVRB and CARS, compared with the experimental lineshapes for Δ*T* = 0 in Fig. 3, unambiguously indicates the presence of electronic resonance in SLG and Bernal FLG. For a given material, the relative weight of the two FWM contributions can be modified by using pulsed excitation and tuning the temporal overlap between PP and SP fields^48^, i.e., changing Δ*T*. The experimentally observed evolution of the FWM signal in FLG as a function of PP-SP delay in Fig. 3 has a trend similar to that shown in Fig. 4e, f as function of *η*_NRVB_/*η*_CARS_, validating the resonance-dominated scenario.

A more quantitative picture can be derived from Eqs. (3) and (4), where the PP and SP temporal profiles are taken into account, matching those retrieved from the experimentally measured autocorrelation (Fig. 2).

As model parameters for the FLG we use the experimental SR $\tilde \nu _{ca} = 1580\,{\mathrm{cm}}^{ - 1}$, with fitted *τ*_*ca*_ = 1.1 ± 0.1 ps^10,45^ (corresponding to FWHM(G) = 10 cm^−1^) and *τ*_*da*_ = *τ*_*ba*_ = *τ*_*ea*_ = 10 ± 2fs in agreement with the value measured for SLG^47^. The ratio between NVRB and CARS contributions $\frac{{\eta _{{\mathrm{CARS}}}}}{{\eta _{{\mathrm{NVRB}}}}} = (3.0 \pm 0.7) \times 10^{ - 5}$ is obtained by fitting the data in Fig. 3 with Eqs. (1), (19), and (20). The resulting spectra (colored dashed lines in Fig. 3), evaluated by tuning only the PP-SP delays, are in good agreement with the experimental data, with $\frac{{\eta _{{\mathrm{CARS}}}}}{{\eta _{{\mathrm{NVRB}}}}}$ as the only adjustable parameter. This ratio, combined with Eqs. (5) and (6), allows us to extract the ratio between the third-order nonlinear susceptibilities for CARS and NVRB, $\frac{{|\chi _{{\mathrm{CARS}}}^{(3)}|}}{{|\chi _{{\mathrm{NRVB}}}^{(3)}|}}\sim 1.3$, at the G-phonon resonance.

The dependence of our spectral profiles on Δ*T* indicates that the peculiar FWM lineshapes for SLG and FLG originate from the interference between two electronically resonant radiation–matter interactions (NVRB and CARS) rather than from a matter-only Hamiltonian coupling the electronic continuum and a discrete phonon state (implying a resonance between the corresponding energies), resulting in the Fano resonance^61^ suggested in ref. ^36^.

### Coherent vibrational imaging

In the electronically nonresonant case, CARS provides access to the real part^23^ of *χ*^(3)^. However, due to the dispersive nature of the *χ*^(3)^ real part^23^, it distorts the phonon lineshapes^3^, unlike SR. In SLG and FLG the FWM signal arises from the imaginary (non dispersive) CARS susceptibility, and it is amplified by its NVRB (third term in Eq. (8)). Then, the signal can be used for vibrational imaging, unlike the nonresonant case, for which the real part of the CARS susceptibility is involved, generating spectral distortions. Thus, coherent vibrational imaging can be performed without suppressing the NVRB, achieving signal levels as large as those of FWM, while preserving the Raman information.

The vibrationally resonant contribution *I* can be isolated by subtracting from the *I*_2_ FWM signal at $\tilde \nu _2 - \tilde \nu _{\mathrm{P}}\sim \tilde \nu _{\mathrm{G}}$, the NRVB obtained by linear interpolation of the spectral intensities measured at the two frequencies at the opposite sides of vibrational resonance

9 $$I = I_1 - I_2 + \frac{{\tilde \nu _2 - \tilde \nu _1}}{{\tilde \nu _3 - \tilde \nu _1}}(I_3 - I_1){,}$$

where the indexes *i* = 1, 2, 3 refer to data at $\tilde \nu _1 = \tilde \nu _{\mathrm{P}} + 1545\,{\mathrm{cm}}^{ - 1}$, $\tilde \nu _2 = \tilde \nu _{\mathrm{P}} + 1576\,{\mathrm{cm}}^{ - 1}$, $\tilde \nu _3 = \tilde \nu _{\mathrm{P}} + 1607\,{\mathrm{cm}}^{ - 1}$ (i.e., with $\tilde \nu _2$ near to the G-phonon frequency and $|\tilde \nu _{1,3} - \tilde \nu _{\mathrm{G}}|$ greater than two half-widths at half maximum of the measured profiles, as shown in Fig. 3).

**Fig. 5:**
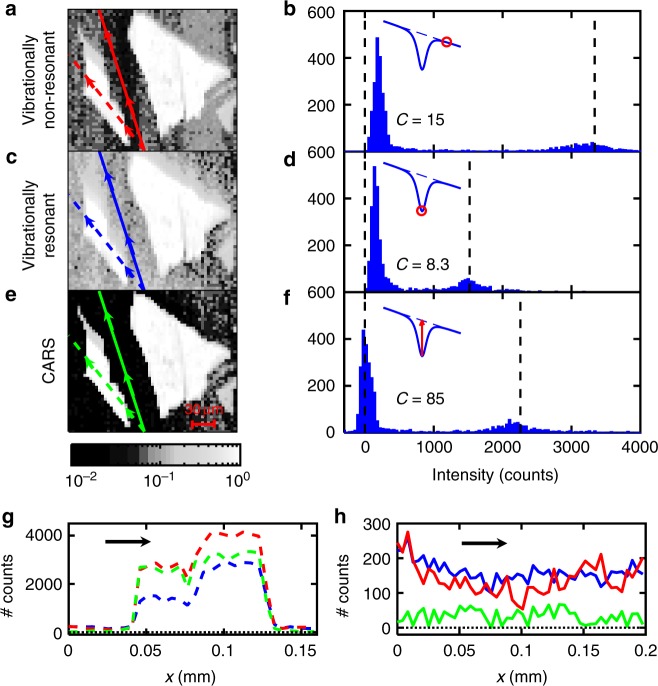
FLG nonlinear optical microscopy. Nonlinear optical images of FLG measured under **a** nonvibrationally resonant *λ*_S_ at 891.5 nm and **c** resonant *λ*_S_ at 894 nm and Δ*T* = 1.7 ps. **e** CARS image of two FLG flakes, obtained by the spectral dip (see Eq. (9)). **b**, **d**, **f** Intensity histograms of (**a**, **c**, **e**). The corresponding contrast *C* is also reported. The black dashed lines represent the colormap boundaries of (**a**, **c**, **e**). **g**, **h** Intensity profiles along the scanning paths in and out of a FLG flake as highlighted in (**a**, **c**, **e**) by dashed and full lines, respectively

This combination of electronically resonant NVRB and CARS nonlinear responses gives CARS images (i.e., retaining vibrational sensitivity) with signal intensities comparable to those of NVRB, for which sub-ms pixel dwell times have been demonstrated with the use of a point detector, e.g., a photomultiplier^2^. In our case, the images in Fig. 5 are obtained with a pixel dwell time ~200 ms using a Si-charge-coupled device array, aiming at a complete spectral characterization, and scanning the sample at fixed Δ*T* with stepper-motor translation stages.

Figure 5a–c displays nonlinear optical images measured at two different *ω*_S_, corresponding to vibrationally nonresonant and resonant conditions. Extracting for each image pixel *I*_1_ (Fig. 5a), *I*_2_ (Fig. 5c), and *I*_3_, required for Eq. (9), we obtain an image with suppression of the signal not generated by FLG, as in Fig. 5e.

To obtain a quantitative comparison of the different images, we plot the pixel intensity histogram in Fig. 5b, d, f. This gives a bimodal distribution: one peak corresponds to the most intense pixels, associated with FLG (*I*_g_) and the other is related to the weaker substrate signal (*I*_s_). The ability to discriminate sample from substrate can be quantified in terms of 1) *I*_g_ compared to *I*_s_, evaluated as the difference *I*_g_ − *I*_s_, and 2) the proximity of *I*_s_ to *I* = 0 in the histogram (dashed black line in Fig. 5b, d, f). These two features can be quantified by the contrast in order to compare the images: $C = \frac{{\overline I _{\mathrm{g}} - \overline I _{\mathrm{s}}}}{{\overline I _{\mathrm{s}}}}$, where $\overline I _{\mathrm{g}}$ and $\overline I _{\mathrm{s}}$ are the mean FLG and substrate intensities, corresponding to the local maxima in the histograms in Fig. 5b, d, f. In Fig. 5g, h, we plot the intensity profiles along two scanning paths, one inside (dashed) and the other adjacent to (full line) the FLG flake.

Comparing the three histograms (Fig. 5b, d, f), the vibrationally off resonant FWM image (NVRB only, Fig. 5b) has the highest $\overline I _{\mathrm{g}}$. The visibility of the flakes is limited by the noise of the detector and by the substrate *χ*^(3)^. NVRB, lacking vibrational specificity, can also originate from the glass substrate outside the FLG flake $\left( {\overline I _{\mathrm{s}} \gg 0} \right)$, as indicated by the scanning profile in Fig. 5h (red line). This may become a critical limitation in those substrates with *χ*^(3)^ much larger than Si (|*χ*^(3)^|~2.5 × 10^−10^ e.s.u.^33^), such as Au (*χ*^(3)^ = 4 × 10^−9^ e.s.u.^62,63^). Similarly, the vibrationally resonant FWM, *I*_2_, originating from concurrent CARS and NVRB processes (Fig. 5d), has a $\bar I_{\mathrm{s}}\gg 0$, related to NRVB. The depth of the FWM dip (Fig. 5f) is related to the CARS signal intensity, and its vibrational sensitivity brings a substantial contrast increase, as demonstrated by the close-to-zero average value of the (green) scanning profile in Fig. 5h.

In summary, by using an experimental time-delayed FWM scheme, CARS peaks equivalent to those of SR were obtained from graphene. By explaining the physical mechanism responsible for the FWM signal, we demonstrated that the spectral response can be described in terms of joint CARS and NVRB contributions concurring to the overall signal. Unlike nonresonant FWM, where dispersive lineshapes hamper vibrational imaging of biological systems, the resonant nature of FWM in graphene, which can be traced back to its peculiar electronic properties, mixes CARS and NVRB, resulting in Lorentzian profiles which are either peaks or dips depending on their relative strength. We also demonstrated that CARS can be used for vibrational imaging with contrast equivalent to spontaneous Raman microscopy and signal levels as large as those of the third-order nonlinear response.

## Methods

### Third-order response of SLG and FLG

The third-order response for SLG and FLG can be obtained from the third-order polarization^20,64^

10 $$\begin{array}{*{20}{l}} {P^{(3)}(t)} \hfill & \propto \hfill & {N\mathop {\int}\limits_0^\infty d \tau _3\mathop {\int}\limits_0^\infty d \tau _2\mathop {\int}\limits_0^\infty d \tau _1{\cal{E}}(t - \tau _3)} \hfill \\ {} \hfill & {} \hfill & { \times {\cal{E}}(t - \tau _2 - \tau _3){\cal{E}}(t - \tau _1 - \tau _2 - \tau _3)S^{(3)}(\tau _1,\tau _2,\tau _3)} \hfill, \end{array}$$

where *N* is the number of scatterer. *S*^(3)^(*τ*_1_, *τ*_2_, *τ*_3_) may be expressed as^4,20^

11 $$\begin{array}{*{20}{l}} {S^{(3)}(\tau _1,\tau _2,\tau _3)} \hfill & \propto \hfill & {\left( i \right)^3Tr\left\{ {\mu (\tau _1 + \tau _2 + \tau _3)} \right.} \hfill \\ {} \hfill & {} \hfill & {\left. { \times [\mu (\tau _1 + \tau _2),[\mu (\tau _1),[\mu (0),\rho ( - \infty )]]]} \right\}} \hfill, \end{array}$$

and ${\cal{E}}(t)$ is the total electric field on the sample

12 $${\cal{E}}(t) = \mathop {\sum}\limits_{i = P,S} {[E_i(t,{\mathrm{\Delta }}t_i) + c.c.]} = \mathop {\sum}\limits_{i = P,S} {[\hat A_i(t,{\mathrm{\Delta }}t_i)e^{ - i\omega _it} + c.c.]}{.}$$

Dispersion effects induce a frequency chirp on ultrashort pulses, i.e., a time dependence of the instantaneous frequency of the optical pulse. The impact of this on the FWM signal can be evaluated by modifying pump and probe spectral profiles as: $A_{{\mathrm{S}}/{\mathrm{P}}}(\omega ,C) = A_{{\mathrm{S}}/{\mathrm{P}}}(\omega )e^{( - iC\omega ^2)}$, where *C* is the group delay dispersion and *A*_S/P_ indicates the PP/SP spectral envelope, i.e., the amplitude of the Fourier transform of the envelope of the electric field of the pulse^65^. The corresponding effect on the CARS profile is a slight intensity modification, below 5% for chirp as large as 10^4^ fs^2^. We note that the chirp introduced by the transmission optics employed in our experiment (1 cm thick PPLN crystal and ~5 cm glass) is^66^ ~6000 fs^2^. Hence the dispersion effect is negligible. Consider a SP at Δ*t*_*S*_ = 0 with Δ*T* = Δ*t*_P_. The energy level diagrams in Fig. 1 schematically illustrate the CARS and NVRB processes^20^

13 $$\begin{array}{*{20}{l}} {P_{{\mathrm{CARS}}}^{(3)}(t, \Delta t)} {\hskip-6pt}\hfill & \propto \hfill & {\hskip-6pt}{\left( i \right)^3n_{{\mathrm{CARS}}}\mu _{ba}\mu _{cb}\mu _{cd}\mu _{ad}\mathop {\int}\limits_0^\infty d \tau _3\mathop {\int}\limits_0^\infty d \tau _2\mathop {\int}\limits_0^\infty d \tau _1} \hfill \\ {} \hfill & {} \hfill & {}{ \times A_{\mathrm{P}} (t - \tau _1 - \tau _2 - \tau _3,{\mathrm{\Delta }}t)A_{\mathrm{S}}^ \ast (t - \tau _2 - \tau _3 ,0)A_{\mathrm{P}}(t - \tau _3,{\mathrm{\Delta }}t)} \hfill \\ {} \hfill & {} \hfill & {}{ \times e^{ - i\omega _{\mathrm{P}}(t - \tau _1 - \tau _2 - \tau _3,{\mathrm{\Delta }}t)}e^{ + i\omega _{\mathrm{S}}(t - {\tau _2} - {\tau _3} )}e^{ - i\omega _{\mathrm{P}}(t - \tau _3)} } \hfill \\ {} \hfill & {} \hfill & {}{ \times e^{ - i\bar \omega _{ba}\tau _1}e^{ - i\bar \omega _{ca}\tau _2}e^{ - i\bar \omega _{da}\tau _3},} \hfill \end{array}$$

14 $$\begin{array}{*{20}{l}} {P_{{\mathrm{NVRB}}}^{(3)}(t, \Delta T)} \hfill & \propto \hfill & {\left( i \right)^3n_{{\mathrm{NVRB}}}|\mu _{ea}|^4{\int}_0^\infty d\tau _3{\int}_0^\infty d\tau _2{\int}_0^\infty d\tau _1} \hfill \\ {} \hfill & {} \hfill & { \times A_{\mathrm{P}} (t - \tau _1 - \tau _2 - \tau _3, \Delta t)A_{\mathrm{P}}(t - \tau _2 - \tau _3, \Delta t)} \hfill \\ {} \hfill & {} \hfill & { \times A_{\mathrm{S}}^ \ast (t - \tau _3,0) e^{ - i\omega _{\mathrm{P}}(t - \tau _1 - \tau _2 - \tau _3)}e^{ - i\omega _{\mathrm{P}}(t - \tau _2 - \tau _3)}} \hfill \\ {} \hfill & {} \hfill & {\times e^{ + i\omega _{\mathrm{S}}(t - \tau _3)}e^{ - i\bar \omega _{ea}\tau _1}e^{ - i\bar \omega _{ea}\tau _2}e^{ - i\bar \omega _{ea}\tau _3}}, \hfill \end{array}$$

where $$\bar \omega _{ij} = \omega _i - \omega _j - i\gamma _{ij}$$.

By Fourier transform, the frequency dispersed signal can be expressed as

15 $$P^{(3)}(\omega ) = \mathop {\int}\limits_{ - \infty }^\infty {P^{(3)}} (t)e^{i\omega t}dt.$$

In order to reduce the computational effort to calculate Eqs. (13) and (14), we also write the pulse fields in terms of their Fourier transforms, obtaining

16 $$\begin{array}{l}P_{{\mathrm{CARS}}}^{(3)}(\omega ) \propto \eta _{{\kern 1pt} {\mathrm{CARS}}}\left( i \right)^3\mathop {\int}\limits_{ - \infty }^{ - \infty } d t\,e^{i\omega t}\mathop {\int}\limits_0^\infty d \tau _3\mathop {\int}\limits_0^\infty d \tau _2\mathop {\int}\limits_0^\infty d \tau _1 \\ \times \mathop {\int}\limits_{ - \infty }^\infty d \omega _1\mathop {\int}\limits_{ - \infty }^\infty d \omega _2\mathop {\int}\limits_{ - \infty }^\infty d \omega _3\hat A_{\mathrm{P}}(\omega _1,\Delta T)\hat A_{\mathrm{S}}^ \ast (\omega _2,0) \hat A_{\mathrm{P}}(\omega _3,\Delta T) \\ \times e^{ - i(\omega _{\mathrm{P}} + \omega _1)(t - \tau _1 - \tau _2 - \tau _3)}e^{ + i(\omega _{\mathrm{S}} + \omega _2)(t - \tau _2 - \tau _3)}\\ \times e^{ - i(\omega _{\mathrm{P}} + \omega _3)(t - \tau _3)}e^{ - i\bar \omega _{ba}\tau _1}e^{ - i\bar \omega _{ca}\tau _2}e^{ - i\bar \omega _{da}\tau _3},\end{array}$$

where *η*_CARS_ = *n*_CARS _*μ*_*ba *_*μ*_*cb *_*μ*_*cd *_*μ*_*ad*_. In this way all the temporal integrals can be solved analytically

17 $$\begin{array}{l}P_{{\mathrm{CARS}}}^{(3)}(\omega , \Delta T) \propto - \eta _{{\mathrm{CARS}}}\mathop {\int}\limits_{ - \infty }^\infty d \omega _1\mathop {\int}\limits_{ - \infty }^\infty d \omega _2\mathop {\int}\limits_{ - \infty }^\infty d \omega _3 \\ \times \delta (\omega - 2\omega _{\mathrm{P}} + \omega _{\mathrm{S}} + \omega _1 - \omega _2 + \omega _3)\\ \times \frac{{\hat A_{\mathrm{P}}(\omega _1,\Delta t)\hat A_{\mathrm{S}}^ \ast (\omega _2,0)\hat A_{\mathrm{P}}(\omega _3,\Delta t)}}{{(\omega _P + \omega _1 - \bar \omega _{ba})(\omega _P - \omega _S + \omega _1 - \omega _2 - \bar \omega _{ca})(2\omega _{\mathrm{P}} - \omega _{\mathrm{S}} + \omega _1 - \omega _2 + \omega _3 - \bar \omega _{da})}},\end{array}$$

using the energy conservation, represented by the delta distribution

18 $$\delta (\omega - 2\omega _{\mathrm{P}} + \omega _{\mathrm{S}} - \omega _1 + \omega _2 - \omega _3) = \mathop {\int}\limits_{ - \infty }^\infty {e^{i(\omega - 2\omega _{\mathrm{P}} + \omega _{\mathrm{S}} - \omega _1 + \omega _2 - \omega _3)t}} ,$$

the *ω*_2_ integral can be simplified

19 $$\begin{array}{l}P_{{\mathrm{CARS}}}^{(3)}(\omega ,{\mathrm{\Delta }}T) \propto - \eta _{{\mathrm{CARS}}}{\int}_{ - \infty }^\infty d\omega _1{\int}_{ - \infty }^\infty d\omega _3\\ \times \frac{{\hat A_{\mathrm{P}}(\omega _3,\Delta T)\hat A_{\mathrm{P}}(\omega _1,\Delta T)\hat A_{\mathrm{S}}^ \ast (2\omega _{\mathrm{P}} - \omega _{\mathrm{S}} - \omega + \omega _3 + \omega _1,0)}}{{\left( {\omega _{\mathrm{P}} + \omega _3 - \bar \omega _{ba}} \right)\left( {\omega - \omega _{\mathrm{P}} - \omega _1 - \bar \omega _{ca}} \right)\left( {\omega - \bar \omega _{da}} \right)}}.\end{array}$$

In a similar way, using *η*_NVRB_ = *n*_NVRB_|*μ*_*ea*_|^4^, Eq. (14) can be written as

20 $$\begin{array}{l}P_{{\mathrm{NRVB}}}^{(3)}(\omega ,\Delta T) \propto - \eta _{{\mathrm{NVRB}}}{\int}_{ - \infty }^\infty d\omega _1{\int}_{ - \infty }^\infty d\omega _2\\ \times\frac{{\hat A_{\mathrm{P}}(\omega _1,\Delta T)\hat A_{\mathrm{P}}(\omega _2,\Delta T)\hat A_{\mathrm{S}}^ \ast (2\omega _{\mathrm{P}} - \omega _{\mathrm{S}} - \omega + \omega _1 + \omega _2,0)}}{{\left( {\omega _{\mathrm{P}} + \omega _1 - \bar \omega _{ea}} \right)\left( {2\omega _{\mathrm{P}} + \omega _1 + \omega _2 - \bar \omega _{ea}} \right)\left( {\omega - \bar \omega _{ea}} \right)}}.\end{array}$$

## Data Availability

All relevant data and Matlab codes are available from the authors.
